# Pyrolysis of Pine Wood in the Presence of Boron–Nitrogen Compounds

**DOI:** 10.3390/ma16196353

**Published:** 2023-09-22

**Authors:** Irina Stepina, Yulia Zheglova

**Affiliations:** 1 Department of Building Materials Science, National Research Moscow State University of Civil Engineering, Yaroslavskoe sh. 26, 129337 Moscow, Russia; 2 Department of Information Systems, Technologies and Automation in Construction, National Research Moscow State University of Civil Engineering, Yaroslavskoe sh. 26, 129337 Moscow, Russia; jeglovayug@mgsu.ru

**Keywords:** pine wood, boron–nitrogen compounds, thermal analysis, pyrolysis, nitrogen atmosphere, correlation and regression analysis

## Abstract

The actuality of this research is determined by the intensification of new ways of processing woody biomass. This requires revealing the impact of various physicochemical factors on the thermal degradation of wood biopolymers. Boron–nitrogen surface modifiers are used for wood antisepsis and we decided to check their effect on flammability. The aim of the research was to evaluate the flame retardant effect of boron–nitrogen surface modifiers of wood in an inert atmosphere (nitrogen was used). The evaluation was carried out by thermal analysis of modified and the control pine wood samples. The thermal analysis included thermogravimetry, differential scanning calorimetry and kinetic parameters of thermal degradation. It was found that the flame retardant effect of boron–nitrogen wood surface modifiers was not significantly pronounced in the nitrogen atmosphere. The mechanism of the flame retardant effect of boron–nitrogen compounds is reduced to “shielding” of the surface and increasing the proportion of carbonized residue. On the basis of correlation–regression analysis of kinetic parameters of wood thermodestruction in a nitrogen atmosphere, mathematical models of activation energy dependence on conversion were obtained and substantiated. The developed models can be further applied to calculate the predicted value of wood activation energy in the nitrogen atmosphere at any conversion value.

## 1. Introduction

The investigation into the thermal characteristics of polymers derived from plants, facilitated by modern techniques such as thermogravimetry and differential scanning calorimetry, has garnered substantial interest. The allure lies in the prospect of unveiling invaluable insights into the thermal stability of distinct components within tree biomass. Such studies hold the key to unraveling the intricate relationship between the chemical composition of these components, particularly the ratios of principal constituents, and the defining features of their thermal decomposition. As novel avenues for wood biomass processing gain momentum, it becomes imperative to delve into the influence of diverse physicochemical factors on the process of thermal degradation of wood biopolymers. In this pursuit, thermogravimetry emerges as a befitting methodology for examining the pyrolytic behavior exhibited by lignocellulosic materials. This approach enables the determination of a myriad of characteristics, including the temperature range encompassing pivotal stages of thermal decomposition, the magnitude of mass loss encountered at corresponding degradation thresholds, the temperature at which maximum mass dissipation occurs, and various other pertinent parameters [1,2,3]. Delving deeper, comprehensive thermal analysis emerges as an indispensable tool, serving as an intellectual beacon, illuminating the trajectory of underlying reactions intricately involved in this process, while concurrently unveiling the precise composition of resulting reaction products. From a practical standpoint, grasping the nuances of thermal decomposition in woody biomass holds paramount importance, as it lays the foundation for evaluating thermal stability and its consequential influence on the yield and properties of thermolysis products.

In the pyrolysis of cellulose, the primary constituent of wood composites, two competing paths of thermal degradation reactions become evident. Dehydration reactions involve the intramolecular transformation of cellulose into dehydrocellulose, which subsequently decomposes into CO_2_, CO, H_2_O, and carbon. Simultaneously, depolymerization encompasses the breaking of bonds in the primary macromolecular chains, resulting in the conversion of cellulose into resin, primarily levoglucosan—a volatile product serving as a key fuel in gas-phase combustion [4]. Experimental results reveal the formation of a viscous oil-like residue in addition to gaseous and volatile products. From this residue, a solid material, termed levoglucosan, is isolated, accounting for 45% of the original cellulose weight [5].

Elemental analysis of cellulose’s chemical composition after exposure to heat demonstrates the occurrence of two competing mechanisms of cellulose thermal degradation within distinct temperature ranges. In the initial range of 200 to 280 °C, dehydration reactions prevail. Dehydration enhances the thermal stability of intermediate products of cellulose decomposition while suppressing depolymerization reactions. It leads to the formation of a thermal decomposition intermediate called dehydrocellulose. The yield of levoglucosan from cotton cellulose reaches 47%, whereas from dehydrocellulose, it does not exceed 9.3%. Variations in levoglucosan yield can be attributed to the wide range of CH_2_OH group conformers present in the cellulose sample [6].

In the subsequent temperature range of 280 to 400 °C, depolymerization reactions take center stage. Cellulose macromolecules break down via 1,4-β-glucoside bonds, followed by isomerization of monomers into levoglucosan molecules [6]. The activation energies for dehydration and depolymerization processes are measured at 146.5 kJ/mol and 188.3–230.2 kJ/mol, respectively [6]. Notably, when cellulose pyrolysis occurs at high temperatures or under rapid heating rates, bonds within the main chain and at the ends of the macromolecule break, leading to depolymerization [7].

Upon heating cellulose above 275 °C, intense decomposition takes place, resulting in the formation of gaseous and liquid products along with the release of heat. The release of these products predominantly ceases at temperatures of 400–450 °C, leaving behind coke as the final residue [8]. While envisioning the reaction equation for complete cellulose pyrolysis solely via an intramolecular mechanism, the final products consist of carbonized residue and water. Notably, in practice, thermal decomposition of cellulose-based materials yields levoglucosan as a significant decomposition product, along with gaseous byproducts such as H_2_O, CO, CO_2_, H_2_, and a variety of saturated and unsaturated hydrocarbons. Additionally, studies reveal the presence of alcohols, ketones, and aldehydes within the resinous residue [8]. Given the flammability and combustibility of these substances, cellulose-based materials pose a relatively high fire hazard.

To mitigate the flammability of cellulose, it is crucial to suppress bond-breaking processes between the structural elements of macromolecules while enhancing dehydration processes and the yield of carbonized residue. Achieving these objectives involves maintaining low heating rates and avoiding elevated temperatures to effectively suppress depolymerization processes. Previous studies [9] postulated that the greatest effect of chemical modification is achieved by targeting primary alcohol groups at the sixth carbon atom. By modifying these groups, the formation of levoglucosan, the most combustible decomposition product, in heat-exposed modified glucose residues can be prevented. This is accomplished by replacing the primary hydroxyl group with alternative functional groups. For instance, monocarboxyl cellulose containing 15.2% CHO groups instead of CH_2_OH groups exhibits reduced thermostability compared to original cellulose. The main stage of thermal decomposition in these modified materials occurs at temperatures of 170–350 °C and 250–370 °C, respectively. Chemical modification involving the replacement of primary hydroxyl groups with COOH groups eliminates the possibility of depolymerization with the formation of levoglucosan [9].

Chemical modification offers a pathway to obtaining both easily flammable and hardly flammable materials [10,11,12,13,14]. For instance, cellulose trinitrate, an explosive and flammable substance containing the NO_2_ group, exhibits a decomposition temperature of 120–140 °C, while cellulose triacetate, a more heat-resistant product, decomposes at temperatures of 220–230 °C and releases acetic acid [15]. Two pivotal approaches to impart fire resistance to cellulose fibers have been proposed: (1) impregnation of fibers with solutions of reactive flame retardants, incorporating them into the spinning solution to confer flame retardant properties to viscose fibers; and (2) application of esterification, alkylation, or grafted polymerization methods to attach flame retardants to cellulose [16,17,18,19,20].

In the context of lignin pyrolysis, as temperatures rise above 150 °C, an early stage of primary condensation commences, driven by the direct involvement of hydroxyl groups, particularly those found in the benzyl alcohol propane chain. These groups foster the formation of interlinks and new double bonds within the propane chain, with guaiacyl moieties of the lignin structure exhibiting the highest activity in this process [21,22,23]. Concurrently, an alternative reaction takes place, involving the breaking of ether aryl–alkyl bonds, which are the least heat resistant. This process initiates at 150 °C and peaks at 300 °C. The thermal destruction of these bonds follows a heterolytic decomposition mechanism, particularly hydrolytic in nature, as confirmed by experimental investigations on methylated alkali lignin’s thermodegradation [24,25]. Regarding cyclic structures in lignin based on diisoeugenol, their thermostability lies within the range of 400–420 °C, indicating the presence of initial precursors for the formation of polyaromatic structures during lignin’s heat treatment above 400–450 °C. The methoxyl group in lignin is also heat resistant, exhibiting no signs of decomposition until temperatures reach 350 °C. Demethylation reactions only occur beyond this threshold [26,27].

Previously published studies [28,29,30] have demonstrated that boron–nitrogen treatments can induce changes in the chemical composition of the lignocarbohydrate complex found within cell walls. The interaction between mono- and diethanolamine(N→B)-trihydroxyborates and cellulose occurs through hydroxyl groups situated at the sixth atom of the glucopyranose ring [28]. Furthermore, it has been established that hydroxyl groups in lignin actively partake in esterification reactions when interacting with boron–nitrogen agents [30]. In light of these findings, this study aims to evaluate the flame-retardant effect of boron–nitrogen modifiers on wood surfaces by analyzing the thermal properties of the modified samples.

## 2. Materials and Methods

Wood modification was conducted utilizing 50% aqueous solutions of boron–nitrogen compounds, namely monoethanolamine(N→B)-trihydroxyborate (hereafter referred to as compound **1**) and diethanolamine(N→B)-trihydroxyborate (hereafter referred to as compound **2**) (Producer NIU MSCU, Moscow, Russia). The surface of air-dry pine wood, with samples containing 8% moisture, underwent treatment via brush application. The modification process transpired at a temperature of 20 °C, with a consumption rate of 150 g/m^2^. The sapwood of freshly cut common pine (Pínus sylvéstris) wood was used as a substrate for modification. Extractive substances were not extracted from the substrate.

Thermal analysis, specifically thermogravimetric analysis (TGA), was carried out on plate-shaped specimens, possessing a thickness of 0.75 mm, carefully extracted from the surface layer of pine wood samples previously subjected to brush application with compounds **1** and **2**. In order to establish a basis for comparison, thermal analysis was also performed on the surface layer of unmodified pine wood samples.

To determine the kinetic parameters associated with the thermal degradation of both modified and unmodified wood (via the integral method), an automated modular thermal analysis system, the “Du Pont-9900” thermal analyzer (Wilmington, DE, USA), was employed. Utilizing the TGA-951 thermoanalyzer, experiments were conducted in dynamic heating mode within a nitrogen atmosphere, gradually ascending to 750 °C, followed by exposure to air (at a flow rate of 50 mL/min) at varying heating rates of 5, 10, and 20 °C/min [31,32].

The acquired thermoanalytical curves were subsequently processed using specialized software, including “File Modification V 1.0”, “General V 1.0”, and TGAKin V 1.0 installed on the TAC “Du Pont 9900” system. Additionally, the Universal Analysis 2000 program from TA Instruments, version V 4.0C (distributed by the Intertec corporation Tampa, FL, USA), played an instrumental role in the analysis.

Employing a graphical approach, the kinetic parameters for the degradation processes were calculated via resolving equation [32]:

(1) E=−(R/0.457)⋅Δ(logβ)/Δ(1/T),

where: *E*—activation energy; 1/*T*—reverse temperature; log *β*—logarithm of heating rate; *R*—universal gas constant.

## 3. Results and Discussion

**Figure 1:**
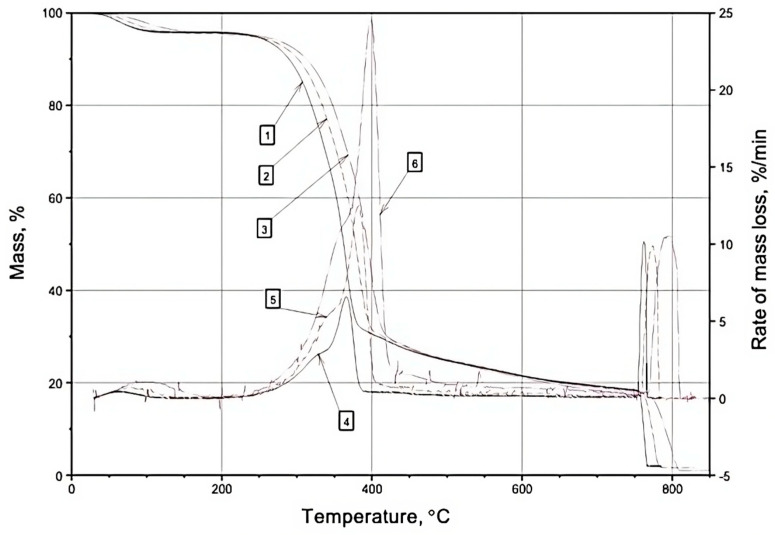
TG(1,2,3) and DTG(4,5,6) curves of samples of unmodified pine wood (nitrogen–air atmosphere): 4—heating rate 5 °C/min; 5—heating rate 10 °C/min; 6—heating rate 20 °C/min.

**Figure 2:**
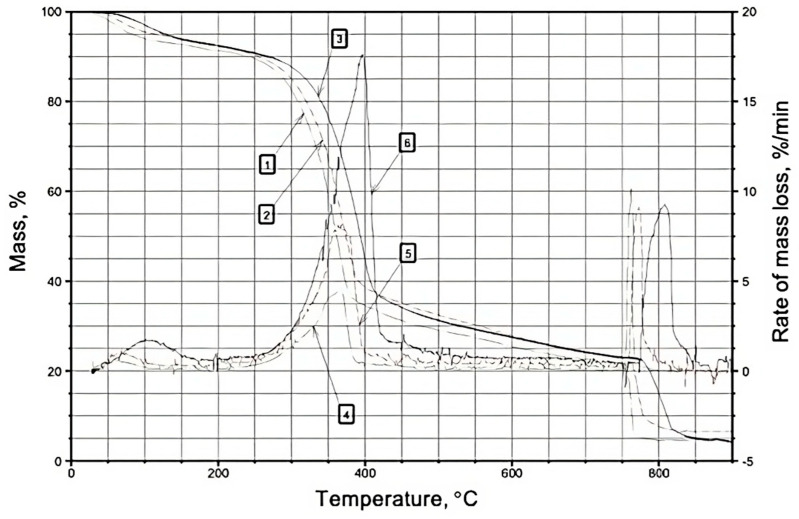
TG(1,2,3) and DTG(4,5,6) curves of samples of the surface layer of pine wood treated with composition **1** (atmosphere nitrogen—air): 4—heating rate 5 °C/min; 5—heating rate 10 °C/min; 6—heating rate 20 °C /min.

**Figure 3:**
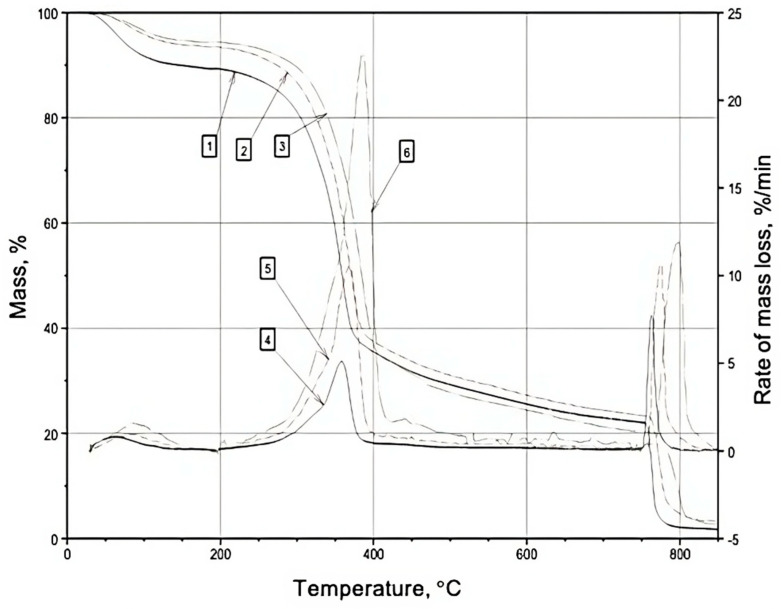
TG(1,2,3) and DTG(4,5,6) curves of samples of the surface layer of pine wood treated with composition **2** (nitrogen–air atmosphere): 4—heating rate 5 °C/min; 5—heating rate 10 °C/min; 6—heating rate 20 °C/min.

In the nitrogen atmosphere, the thermodegradation of wood can be characterized by two distinct stages: the first stage (~30–190 °C) involves the removal of surface adsorption water, while the second stage (~175–500 °C) entails the destruction of wood components, as depicted in Figure 1, Figure 2 and Figure 3 and Table 1, Table 2 and Table 3. During the first temperature interval, the modified wood samples exhibit a higher mass loss (%) compared to the control samples at all heating rates (5, 10, 20 °C/min). This observation aligns with the increased substrate polarity resulting from the modification of wood with boron–nitrogen compounds (components of compounds **1** and **2**), leading to enhanced retention of adsorption water through intermolecular interactions. Consequently, the evaporation of a greater amount of water from the surface of the modified samples results in a higher mass loss compared to native wood.

In the second temperature range (175–500 °C), the destruction of wood components occurs. Across all heating rates, the mass loss (%) and mass loss rate (%/min) of the modified wood samples are lower than those of the control samples, as indicated in Table 1, Table 2 and Table 3. It is well known from the scientific literature [33] that the thermodegradation of wood in a nitrogen atmosphere primarily involves the non-oxidative destruction of molecules. Notably, lignin emerges as the most thermostable component of wood. The degradation of carbohydrate components commences with the cleavage of glycosidic bonds (~200 °C), followed by the breakdown of C-C bonds within the pyranose ring, giving rise to the formation of low-molecular-weight combustible compounds such as levoglucosan, furan, glycol aldehyde, among others [34]. Furthermore, the degradation of cellulose initiates from its least ordered macromolecular regions, where the weakest bonds are broken, leading to an increase in the crystallinity of cellulose during heat treatment up to approximately 200 °C. The modifier molecules, i.e., boron–nitrogen compounds, chemically interact with reactive groups present in the amorphous regions of cellulose, promoting the ordering of its structure [35] and, subsequently, decelerating the thermal degradation of cellulose macromolecules. Steric factors may also contribute to the lower mass loss observed in the modified samples within the nitrogen atmosphere, owing to the prolonged presence of modifier molecules on the wood surface due to the absence of oxidative processes. Notably [36], the vibrational motion of atoms during heating is a known cause of bond rupture within molecules. Therefore, it can be postulated that the voluminous boron–nitrogen compound molecules grafted onto the wood surface partially “shield” the wood components, resulting in reduced vibrational motion of their atoms attributed to spatial considerations. Interestingly, molecules of diethanolamine(N→B)-trihydroxyborane (a component of compound **2**) possess larger sizes compared to molecules of monoethylamine(N→B)-trihydroxyborane (a component of compound **1**). Consequently, wood samples surface-modified with compound **2** display lower mass loss values. Moreover, chemical modification contributes to the enlargement of carbohydrate and lignin fragments, thereby decreasing the yield of low-molecular-weight compounds that constitute the fraction of volatile combustible products.

The kinetic parameters associated with the thermodegradation of wood in a nitrogen atmosphere are presented in Table 4. It is evident that the activation energy of unmodified wood samples slightly decreases with an increasing degree of wood material transformation. Since the thermodegradation of wood entails a continuous temperature rise, with amorphization of cellulose and softening of lignin occurring at higher temperatures (t = 200–280 °C), additional energy is expended, thereby resulting in subsequent thermodecomposition of wood components occurring at lower activation energy values. Beyond a conversion degree of approximately 30%, the activation energy remains virtually unchanged. For the modified wood samples, the activation energy of thermodegradation increases with an increasing degree of conversion. The lower activation energy values observed in the wood samples modified with compositions **1** and **2**, in comparison to the control samples, likely stem from the fact that, at the initial stage, the thermal transformation affects the modifier molecules on the wood surface rather than the wood itself.

At 750 °C, the atmosphere of wood thermal decomposition was altered from nitrogen to air, and the rate of coke oxidation by atmospheric oxygen was examined. The rate of coke oxidation in an air atmosphere, under heating rates of 5, 10, and 20 °C/min, falls within the range of 10–11.9%/min, as indicated in Table 1, Table 2 and Table 3. Across all heating rates, the coke oxidation rate in wood samples modified with composition **2** surpasses that of the native wood. Wood samples surface-modified with composition **1** exhibit a higher coke oxidation rate at a heating rate of 5 °C/min compared to unmodified wood. However, at other heating rates (10, 20 °C/min), the coke oxidation rate is lower than that of the control samples. It is plausible to suggest that the increased rate of mass loss observed in the modified wood samples during the coke oxidation stage, when exposed to atmospheric oxygen, can be attributed to the “burnout” of modifiers. Boron–nitrogen compounds possess lower activation energy in oxidation reactions, resulting in their oxidation occurring prior to that of wood components, and leading to the formation of volatile nitrogen oxides and water vapor [37]. The rapid removal of these volatile oxidation products of boron–nitrogen compounds during the change in atmosphere (t > 750 °C) possibly weakens the top layer of coke, rendering it more susceptible to atmospheric oxygen. This phenomenon can further contribute to an increased rate of mass loss in the modified samples. Lower mass loss values resulting from coke oxidation are observed in wood samples surface-modified with compound **1**, likely due to the structural characteristics of the modifier molecule-monoethanolamine(N→B)-trihydroxyborate (with a lower content of methylene groups). Consequently, the oxidation process generates fewer volatile products compared to the oxidation of components in compound **2**.

By utilizing paired regression analysis, we established a correlation between activation energy and wood conversion in a nitrogen atmosphere. This correlation model enables the prediction of wood activation energy for any conversion value within the range of 0 to infinity. The experimental data supporting this analysis are summarized in Table 4.

**Figure 4:**
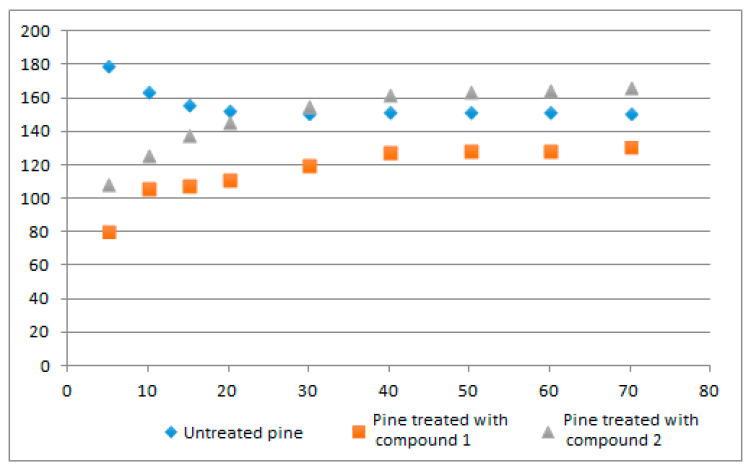
Experimental data correlation field.

We were studying the samples of untreated wood and pine treated by compound **1** or **2**. Prior to conducting the analysis, we constructed correlation fields for each sample type, Figure 4.

These correlation fields (Figure 4) indicated that a linear regression model was not suitable for the available data, necessitating the exploration of non-linear models to determine the degree of dependence.

To build the activation energy-conversion relationship in a nitrogen atmosphere, we considered widely used models such as power, semi-logarithmic, and exponential. In order to estimate the non-linear regression equation, we employed the correlation coefficient as a measure of the strength of the relationship. This coefficient ranges from zero to one. The stronger the relationship between the variables, the higher the value of the coefficient. In addition, we calculated the square of the correlation coefficient, called the determination coefficient. It represents the proportion of variance of the resulting variable y, which was also calculated [38]:

(2) $$\rho_{xy}=\sqrt{1-\frac{\sigma_{ost}^{2}}{\sigma_{y}^{2}}},$$

where $\sigma_{y}^{2}=\frac{1}{n}\sum {\left(y-\bar{y}\right)}^{2}$ is the total variance of the resulting feature *y*, $\sigma_{ost}^{2}=\frac{1}{n}\sum {\left(y-{\hat{y}}_{x}\right)}^{2}$ is the residual variance [38].

The value of this indicator is within $0\leq \rho_{xy}\leq 1$. The closer the correlation value is to one, the closer the relationship of the characteristics under consideration, the more reliable the regression equation.

The square of the correlation coefficient is called the determination coefficient and characterizes the share of the variance of the effective feature *y*, explained by regression, in the total variance of the effective feature [38]:

(3) $$\rho_{xy}^{2}=1-\frac{\sigma_{ost}^{2}}{\sigma_{y}^{2}},$$

We will use the determination coefficient $\rho_{xy}^{2}$ to check the significance of the regression equation in general according to the Fisher *F*-criterion [38]:

(4) $$F=\frac{\rho_{xy}^{2}}{1-\rho_{xy}^{2}}\cdot \frac{n-m-1}{m},$$

where $\rho_{xy}^{2}$ is the determination coefficient, *n* is the number of observations, and *m* is the number of parameters for the variable *x*. The actual value of the *F*-criterion is compared with the table value at the significance level α and the number of degrees of freedom *k*_2_ = *n* − *m* − 1 (for the residual sum of squares) and *k*_1_ = *m* (for the factorial sum of squares) [38].

To evaluate the quality of the non-linear equation, we examined the relative deviations for each observation and calculated the average approximation error using Formula (5).

(5) $$\bar{A}=\frac{1}{n}\sum \left|\frac{y-{\hat{y}}_{x}}{y}\right|\cdot 100\%.$$

The analysis began by proposing a power regression model for the data presented in Table 4. The calculated coefficients resulted in regression equations for untreated pine

*y* = 187.28*x*^−0.056^, (6)

pine treated with compound **1**

*y* = 67.229*x*^0.1662^, (7)

and pine treated with compound **2**.

*y* = 86.645*x*^0.163^. (8)

The analysis results for the power regression model are detailed in Table 5. 

**Figure 5:**
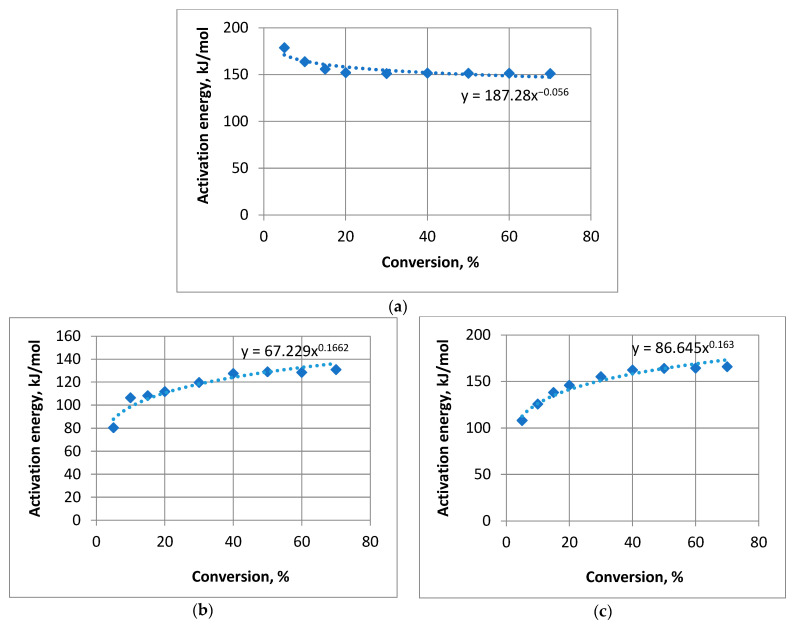
Power regression model: (**a**) untreated pine; (**b**) pine treated with compound **1**; (**c**) pine treated with compound **2**.

Let us depict the initial data and the power regression model in Figure 5:

It was evident that the steppe regression model closely approximated the initial data for both compositions. The calculated values of Fisher’s *F*-criterion significantly exceeded the tabulated value *F_table_* = 5.59 [39]. The average approximation error $\bar{A}$ for all three cases remained below 4%. The determination coefficient is relatively high. For the pine treated by compound **1**, the other factor’s influence (beyond the variation explained by the trait factor) constituted 8.67%. For the pine covered by compound **2**, the value was 5%. However, for the untreated pine, the side factor impact was 22.39%. Therefore, we can make a conclusion that the impact of the other factors on the untreated pine strongly exceeds the one for the treated objects.

Next, we examined the exponential model. The regression equations based on the experimental data in Table 4 were derived as follows: untreated pine

*y* = 165.19*e*^−0.002*x*^, (9)

pine treated with composition **1**

*y* = 95.411*e*^0.0055*x*^, (10)

and pine treated with composition **2**

*y* = 121.9*e*^0.0055*x*^. (11)

The analysis results for the exponential regression model are presented in Table 6. 

**Figure 6:**
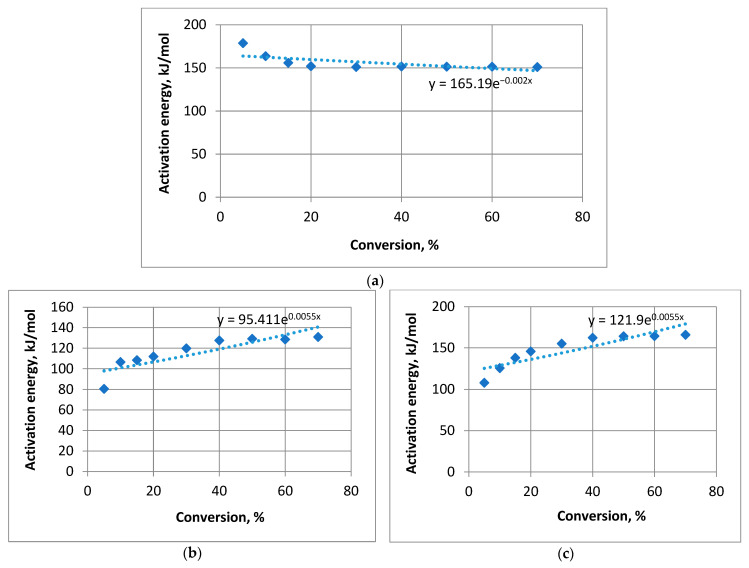
Exponential regression model: (**a**) untreated pine; (**b**) pine treated with compound **1**; (**c**) pine treated with compound **2**.

Figure 6 illustrates the exponential regression model for each composition. 

The calculated values of Fisher’s *F*-criterion exceeded the tabulated value *F_table_* = 5.59 [39], although to a lesser extent compared to the power model. Moreover, the average approximation error for pine treated with compounds 1 and 2 exceeded 5%, indicating larger deviations compared to the power model. The determination coefficient was lower for this model across all samples, indicating a weaker fit. The percentages of other factors were also higher compared to the power model, for the samples filmed by compound **1**—29.84%, by compound **2**—26.98%, and plain wood—58.81%, which is unacceptably large. The resulting percentages for side factors for compounds **1** and **2** significantly exceed the corresponding number values derived by the previous model. As a consequence, we discarded this model for our study.

We then moved on to the semi-logarithmic regression model. The regression equations for each composition were as follows: untreated pine

*y* = −9.194ln(*x*) + 185.97, (12)

pine covered with compound **1**

*y* = 17.809ln(*x*) + 58.627, (13)

and pine covered with compound **2**

*y* = 22.565ln(*x*) + 75.143. (14)

The analysis results for the semi-logarithmic regression model are presented in Table 7. 

**Figure 7:**
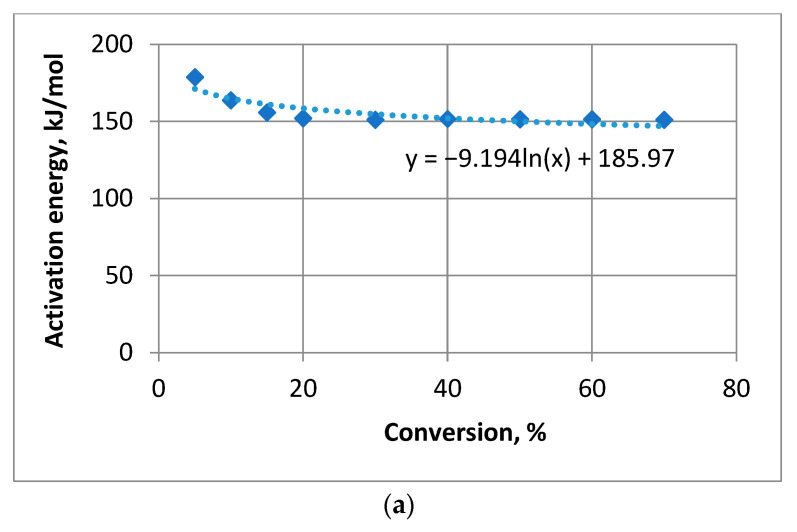

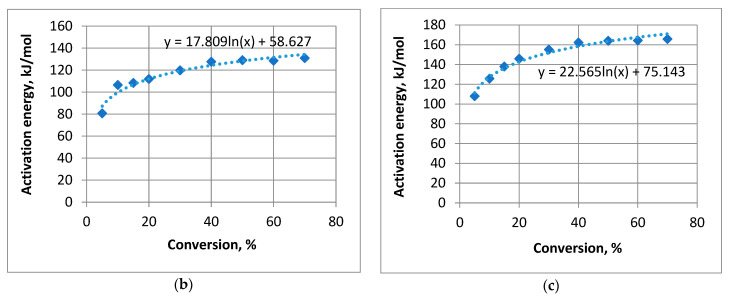
Semi-logarithmic regression model: (**a**) untreated pine; (**b**) pine treated with compound **1**; (**c**) pine treated with compound **2**.

Figure 7 illustrates the semi-logarithmic regression model for each composition.

The analysis indicated that the semi-logarithmic regression model approximated the raw data well for both compounds. The calculated values of Fisher’s *F*-criterion significantly exceeded the tabulated value *F_table_* = 5.59 [39]. The average approximation error $\bar{A}$ for pine treated with compounds 1 and 2, as well as for the plain wood, remained below 3%, indicating a strong fit. The determination coefficient for pine treated with both compositions was high: for compound **1**, 94.1% is the variation of the trait factor and 5.9% corresponds to other factors; for compound **2**—97.27% is part of the trait factor, while 2.73% is related to others. Those numbers indicate a reliable relationship for filmed wood. However, for untreated pine, the determination coefficient was lower (75.92% for trait factor), indicating a higher percentage (24.08%) of other factors influencing the relationship. This tells us that the power model is more useful for untreated wood, while for compounds **1** and **2** logarithmic model is more precise.

The results of our computations using our models are presented in Table 8, Table 9 and Table 10.

From Table 8 it can be seen that the best model that relates the activation energy of wood in a nitrogen atmosphere and the conversion for untreated pine is a power regression model, since the determination coefficient for this model is closest to **1**, and the average approximation error is the smallest.

In summary, the analysis results indicated that the semi-logarithmic model best approximated the experimental data for pine treated with composition **1** (Table 9). This model exhibited a determination coefficient of 0.941, which closely approached **1**, and the smallest average approximation error. Thus, we confidently concluded that this model reliably represented the relationship between activation energy and conversion.

Table 10 shows that the best model for pine treated with composition **2**, as well as for pine treated with compound **1**, is the semi-logarithmic model, as it has a determination coefficient equal to 0.9727 and an average approximation error of 1.7224, which indicates that this model is reliable.

Based on the analysis, we conclude that the semi-logarithmic model approximates the experimental data for the wood treated with compounds **1** and **2** in an inert atmosphere in the best way. Therefore, this model should be used in further work as a way to evaluate activation energy as a function of conversion, discarding all other models that were considered and analyzed in this article, due to their lower statistical reliability. Also, the analysis confirmed that for untreated pine, the power model should be applied.

In the paper, based on the processing and analysis of experimental data, predictive models were derived and validated to determine a one-to-one correspondence of activation energy from conversion for wood in a nitrogen atmosphere. Regression coefficients were calculated, and their reliability was analyzed, the results obtained are presented in Table 11.

In the obtained equation the variable *x* is activation energy, kJ/mol, *y* is conversion, %.

## 4. Conclusions

In the nitrogen atmosphere, the flame-retardant properties of boron–nitrogen wood surface modifiers exhibit insignificance. It is plausible that the mechanism underlying the flame-retardant effect of boron–nitrogen compounds can be attributed to the act of “shielding” the wood surface while augmenting the proportion of carbonized residue. It may be conjectured that the higher activation energy values observed in wood samples modified with compound **2** stem from the presence of a heterocycle encompassing boron and nitrogen atoms within the molecular structure of diethanolamine(N→B)-trihydroxyborate, which becomes grafted onto the wood surface, distinguished by its remarkable inclination towards carbon formation. The resultant carbonized layer, acting as a reflector for a portion of the heat flux, bestows thermal insulation upon the wood surface treated with compound **2**, consequently demanding greater energy for subsequent thermal degradation of the wood. Probably, the thermal decomposition of wood in a nitrogen atmosphere coincides with the formation of low-molecular-weight products arising from the detachment of functional groups present in carbohydrates and lignin. This process elicits the emergence of multiple bonds and cyclic structures, giving rise to the formation of novel intermolecular linkages and the consequent development of carbonized residue.

As it is well known, the predisposition for carbonization represents an additive property intrinsic to organic molecules. Each constituent group found within the composition of wood’s carbohydrate and lignin molecules contributes uniquely to the generation of coke residue during the process of pyrolysis. Alterations in the chemical composition of the wood surface, as a consequence of its modification with boron–nitrogen compounds, notably through the introduction of groups comprising boron and nitrogen atoms, contribute substantially to the carbonization processes at play.

Mathematical models depicting the dependency of activation energy on conversion were successfully derived and validated through correlation–regression analyses conducted on the kinetic parameters associated with wood’s thermo-degradation in a nitrogen atmosphere. These developed models possess the potential for calculating predicted values of wood activation energy under nitrogen atmospheric conditions across a broad range of conversion values.

## Figures and Tables

**Table 1:** Results of thermal analysis of unmodified pine wood with change of atmosphere (nitrogen up to 750 °C further air).

Characteristic TG Curve	Heating Rate, °C/min		
	5	10	20
Temperature range of destruction, °C	30–175	30–175	30–175
Mass loss location on, %	4.1	4.4	4.3
Maximum temperature, °C	63	77	100
Temperature range of destruction, °C	175–500	175–500	175–500
Mass loss location on, %	71	70.4	71.3
Maximum temperature, °C	366	382	398
Speed (%/min) of coke oxidation	10.2	10.0	10.5

**Table 2:** The results of thermal analysis of samples of the surface layer of pine wood treated with composition **1**.

Characteristic TG Curve	Heating Rate, °C/min		
	5	10	20
Temperature range of destruction, °C	30–150	30–150	30–150
Mass loss location on, %	7.24	5.98	6.85
Maximum temperature, °C	60	67	102
Temperature range of destruction, °C	200–500	200–500	200–500
Mass loss location on, %	63.6	61.5	61.9
Maximum temperature, °C	366	366	396
Speed (%/min) of coke oxidation	10.2	9.05	9.22

**Table 3:** The results of thermal analysis of samples of the surface layer of pine wood treated with composition **2**.

Characteristic TG Curve	Heating Rate, °C/min		
	5	10	20
Temperature range of destruction, °C	30–162	30–182	30–191
Mass loss location on, %	10.3	6.4	5.5
Maximum temperature, °C	60	81	91
Temperature range of destruction, °C	162–500	182–500	191–500
Mass loss location on, %	60.5	62.6	66.6
Maximum temperature, °C	358	368	386
Speed (%/min) of coke oxidation	7.7	10.7	11.9

**Table 4:** Kinetic parameters of wood thermodegradation in nitrogen atmosphere.

Conversion, %	Activation Energy, kJ/mol		
	Untreated Pine	Pine Treated with Compound 1	Pine Treated with Compound 2
5.0	178.8	80.6	108.1
10.0	163.8	106.5	125.7
15.0	155.9	108.3	138.1
20.0	152.1	111.9	145.9
30.0	151.1	119.8	155.2
40.0	151.7	127.6	162.4
50.0	151.5	129.1	164.1
60.0	151.5	128.6	164.4
70.0	151.1	131.0	165.9

**Table 5:** Power regression model analysis data.

	Untreated Pine	Pine Treated with Compound 1	Pine Treated with Compound 2
$\rho_{xy}$	0.88095	0.95565	0.9747
$\rho_{xy}^{2}$	0.7761	0.9133	0.95
*F*	24.2612	73.7094	132.923
$\bar{A}$	1.9813	3.5121	2.4699

**Table 6:** Exponential regression model analysis data.

	Untreated Pine	Pine Treated with Compound 1	Pine Treated with Compound 2
$\rho_{xy}$	0.6796	0.8376	0.8545
$\rho_{xy}^{2}$	0.4619	0.7016	0.7302
*F*	6.0077	16.4563	18.9492
$\bar{A}$	2.9	6.8824	5.7206

**Table 7:** Semi-logarithmic regression model analysis data.

	Untreated Pine	Pine Treated with Compound 1	Pine Treated with Compound 2
$\rho_{xy}$	0.8713	0.97	0.98625
$\rho_{xy}^{2}$	0.7592	0.941	0.9727
*F*	22.073	111.7289	249.2248
$\bar{A}$	2.095	2.7228	1.7224

**Table 8:** Comparative analysis of the considered models for pine without treatment.

Model	Determination Coefficient, $\rho_{xy}^{2}$	Average Approximation Error, $\bar{A}$, %
Power regression model	0.7761	1.9813
Exponential regression model	0.4619	2.9
Semi-logarithmic regression model	0.7592	2.095

**Table 9:** Comparative analysis of the considered models for pine treated with composition **1**.

Model	Determination Coefficient, $\rho_{xy}^{2}$	Average Approximation Error, $\bar{A}$, %
Power regression model	0.9133	3.5121
Exponential regression model	0.7016	6.8824
Semi-logarithmic regression model	0.941	2.7228

**Table 10:** Comparative analysis of the considered models for pine treated with composition **2**.

Model	Determination Coefficient, $\rho_{xy}^{2}$	Average Approximation Error, $\bar{A}$, %
Power regression model	0.95	2.4699
Exponential regression model	0.7302	5.7206
Semi-logarithmic regression model	0.9727	1.7224

**Table 11:** Models that establish the dependence of the activation energy on the conversion of wood in a nitrogen atmosphere.

Substrate Type	Equation
Untreated pine	*y* = 187.28*x*^−0.056^
Pine treated with compound **1**	*y* = 17.809ln(*x*) + 58.627
Pine treated with compound **2**	*y* = 22.565ln(*x*) + 75.143

## Data Availability

Not applicable.
